# AQP3 is regulated by PPARγ and JNK in hepatic stellate cells carrying PNPLA3 I148M

**DOI:** 10.1038/s41598-017-14557-9

**Published:** 2017-11-07

**Authors:** Matteo Tardelli, Francesca V. Bruschi, Thierry Claudel, Veronica Moreno-Viedma, Emina Halilbasic, Fabio Marra, Merima Herac, Thomas M. Stulnig, Michael Trauner

**Affiliations:** 1 Hans Popper Laboratory of Molecular Hepatology, Division of Gastroenterology & Hepatology, Internal Medicine III, Medical University of Vienna, Vienna, Austria; 2 Christian Doppler-Laboratory for Cardio-Metabolic Immunotherapy and Clinical Division of Endocrinology and Metabolism, Internal Medicine III, Medical University of Vienna, Vienna, Austria; 3 Institute of Cancer Research, Department of Medicine I, Comprehensive Cancer Center, Medical University of Vienna, Vienna, Austria; 4 Department of Experimental and Clinical Medicine, University of Florence, Florence, Italy; 5 Clinical Institute of Pathology, Medical University of Vienna, Vienna, Austria

## Abstract

Aquaglyceroporins (AQPs) allow the movement of glycerol that is required for triglyceride formation in hepatic stellate cells (HSC), as key cellular source of fibrogenesis in the liver. The genetic polymorphism I148M of the patatin-like phospholipase domain-containing 3 (PNPLA3) is associated with hepatic steatosis and its progression to steatohepatitis (NASH), fibrosis and cancer. We aimed to explore the role of AQP3 for HSC activation and unveil its potential interactions with PNPLA3. HSC were isolated from human liver, experiments were performed in primary HSC and human HSC line LX2. AQP3 was the only aquaglyceroporin present in HSC and its expression decreased during activation. The PPARγ agonist, rosiglitazone, recovered AQP3 expression also in PNPLA3 I148M carrying HSC. When PNPLA3 was silenced, AQP3 expression increased. In liver sections from patients with NASH, the decreased amount of AQP3 was proportional to the severity of fibrosis and presence of the PNPLA3 I148M variant. In PNPLA3 I148M cells, the blockade of JNK pathway upregulated AQP3 in synergism with PPARγ. In conclusion, we demonstrated profound reduction of AQP3 in HSC carrying the PNPLA3 I148M variant in parallel to decreased PPARγ activation, which could be rescued by rosiglitazone and blockade of JNK.

## Introduction

Hepatic stellate cells (HSC) represent central players in the pathogenesis of liver fibrosis^1,2^. Quiescent HSCs store vitamin A in the liver. However, in response to hepatic injuries, HSCs may undergo activation and transdifferentiate into a highly proliferative and myofibroblast-like phenotype responsible for hepatic fibrogenesis^3,4^.

Lipid content is a crucial factor in HSC pathophysiology^5,6^ and lipogenic activity decreases in parallel with vitamin A content during HSC activation^7^. In a recent study we showed that primary HSC carrying the human genetic variant of PNPLA3 (adiponutrin), known as PNPLA3 I148M, lack peroxisome proliferator-activated receptor gamma (PPARγ, NR1C3) expression and activity^8^, which is closely linked to HSC activation^9^. This PNPLA3 I148M variant has been associated to higher accumulation of fat in liver, steatohepatitis and inflammation, progression to fibrosis/cirrhosis and liver cancer^10,11^. PPARγ, the master regulator of adipogenesis, is a ligand activated nuclear receptor, which is mainly expressed in adipose tissue and plays a crucial role in adipogenesis and energy metabolism. In liver, PPARγ is involved in HSC lipid storage, conveys their quiescence and, is reduced in activated HSCs as a direct effect of JNK activation^9,12^.

Aquaglyceroporins (AQPs) are channel proteins, facilitating glycerol diffusion in cells^13,14^. Glycerol represents the backbone structure for triglyceride synthesis which is a fundamental factor in cell metabolism^15^. In HSC, AQPs expression and function are poorly understood. To our knowledge, only one study showed that changes in AQPs expression and water permeability may increase resistance to apoptosis in activated HSCs, although the metabolic impact of glycerol diffusion in activation and quiescence was not analyzed^16^. Importantly, previous studies revealed repression of AQP3 by LPS exposure in human colon epithelial HT-29 cells^17^ but induction in murine 3T3-L1 adipocytes^18^, in line with AQP3 upregulation by arachidonic acid or prostaglandin in human retinal pigment epithelial cells, already suggesting a role of JNK^19^. Since in PNPLA3 I148M expressing HSCs the JNK/AP-1 pathway is strongly induced^20^, the subsequent PPARγ downregulation, led us to hypothesize that PPARγ could regulate AQPs expression in HSC in connection to the I148M variant^20^.

Therefore, we aimed to uncover (i) which AQPs were expressed in primary hepatic stellate cells, (ii) how AQP expression is regulated at the molecular level and (iii) whether PNPLA3 mutations modulated AQP levels in HSC.

## Results

### AQP3 is the only adequately expressed aquaglyceroporin in human primary HSC and down-regulates during their activation

**Figure 1:**
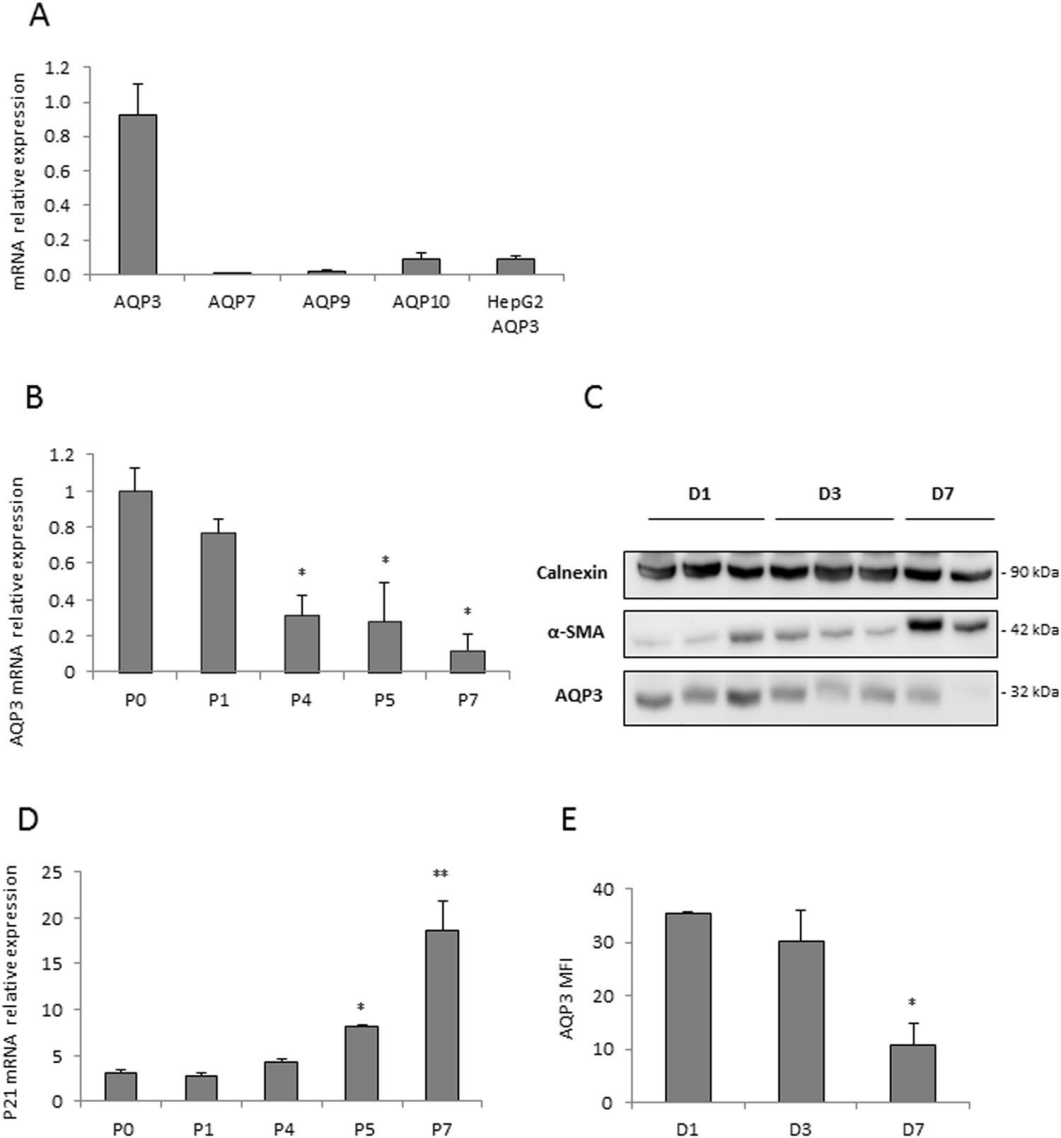
AQP3 is the only aquaporin expressed in human HSCs and its expression decreases proportionally to cells activation and senescence. (**A**) relative mRNA expression of the 4 known aquaglyceroporins in HSC;(**B**) AQP3 mRNA expression in primary HSC decreases with cells passage indicated with P (*p < 0.05 vs P0. p values for P4: p = 0.03; P5: 0.04; P7: 0.02). (**C**) Representative western blot performed in different primary HSCs, categorized according to days (**D**) in cell culture; AQP3 decreases with time and cells activation, calnexin was used as loading control and α-SMA as a marker for HSC activation. (**D**) P21 expression increases with cell passage (P), highlighting HSCs senescence (*p < 0.05, **p < 0.01 compared to P0. p values for P5: p = 0.03; P7: p = 0.009); (**E**) Quantification of flow cytometric indirect staining for AQP3 shows decreased AQP3 expression over time expressed in days (**D**) in accordance to the western blot results (*p = 0.04); gating depicted in Suppl. Figure 1. Means ± SD value of n = 3 independent experiments performed in duplicates.

We explore the expression of all known aquaglyceroporin AQP3, -7, -9 and -10 present in primary HSC expressing the WT PNPLA3 variant by quantitative RT-PCR. Interestingly, only AQP3 was detectable in human primary HSC, but not in hepatocytes (HepG2 cell line) (Fig. 1A). Therefore, we measured its expression in stellate cells during their activation. Over time, there was a profound downregulation of AQP3 paralleled by an increased expression of the senescence marker, p21 (Fig. 1B and D) and the profibrogenic marker α-SMA (not shown). Moreover, protein levels of AQP3 (Fig. 1C) and AQP3 surface staining quantified by a flow cytometric assay (Fig. 1E and Suppl. Figure 1) were reduced. Collectively, these data demonstrated the presence of AQP3 in primary human HSC and its down regulation during HSC activation.

### PPARγ agonist increases AQP3 expression in LX2 cells in association with enhanced lipogenesis and reduced PNPLA3 expression

**Figure 2:**
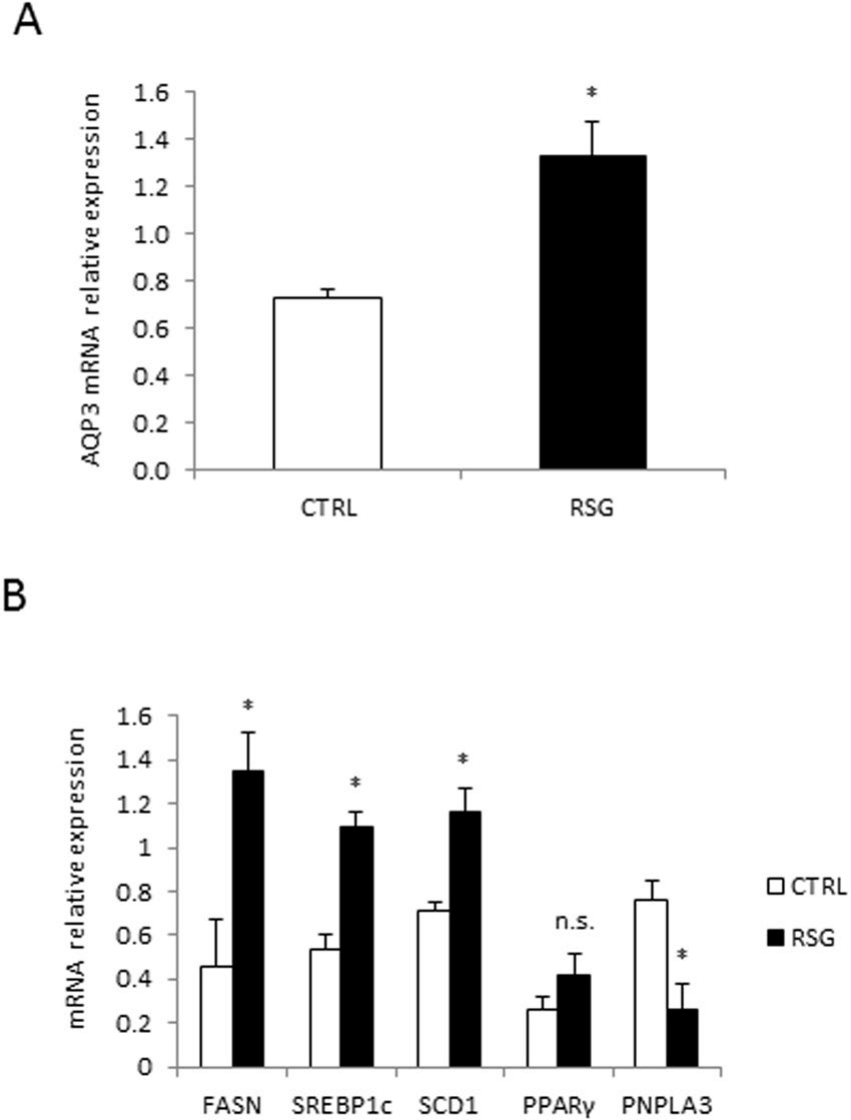
Rosiglitazone stimulates AQP3 expression and lipogenesis in LX2 cells, downregulating PNPLA3 expression. (**A**) AQP3 mRNA expression in LX2 cells treated for 24 h with 0.05 mol/L of rosiglitazone (RSG) compared to control (*p < 0.05);. (**B**) Relative mRNA expression of various key genes of lipid metabolism in LX2 cells treated with RSG versus control (CTRL). *p < 0.05 RSG vs CTRL: *FASN* (p = 0.02), *SREBP1c* (p = 0.04), *SCD1* (p = 0.03), *PPAR*γ (not significant – n.s.) and *PNPLA3* (p = 0.04) genes.

Since AQP3 is a PPARγ target gene^21^, and PPARγ is strongly down-regulated in fully activated HSCs^20^, we further explored whether a PPARγ agonist, rosiglitazone (RSG), may reverse the repression of AQP3 during HSC activation. AQP3 mRNA expression increased after RSG stimulation in LX2 cells (Fig. 2A), as well as lipogenesis (demonstrated by increased gene expression of FASN, SREBP1c, SCD1 and PPARγ) while expression of PNPLA3 was significantly reduced (Fig. 2B). These results demonstrate that the regulation of AQP3 is strongly PPARγ dependent in HSC; to such an extent that administration of a PPARγ agonist up-regulates AQP3 expression.

### AQP3 expression is reduced in PNPLA3 I148M cells and patients but it is restored *in vitro* by the PPARγ agonist rosiglitazone

**Figure 3:**
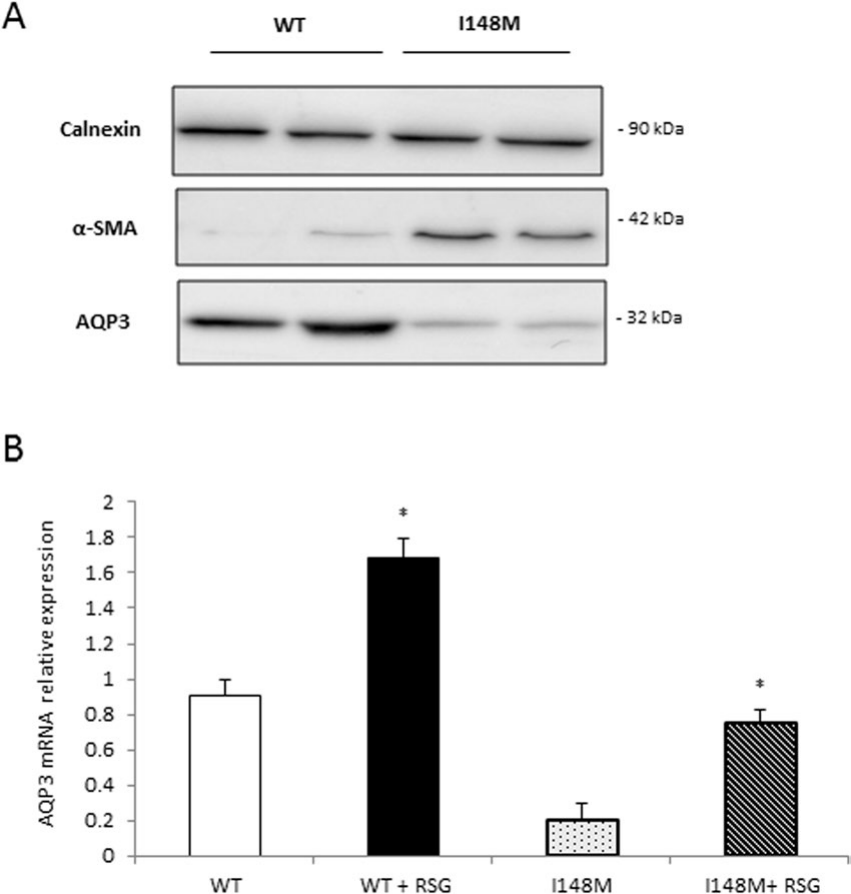
LX2 overexpressing WT and I148M PNPLA3 show lower AQP3 expression which is restored by rosiglitazone treatment. (**A**) Representative western blot of LX2 overexpressing PNPLA3 I148M showed dramatically reduced AQP3 expression compared to WT. (**B**) AQP3 mRNA expression is recovered after treatment with rosiglitazone (RSG) in LX2 overexpressing WT and I148M due to PPARγ induction (*p < 0.05, RSG treated HSC WT vs. WT control and RSG treated HSC I148M vs untreated HSC I148M).

**Figure 4:**
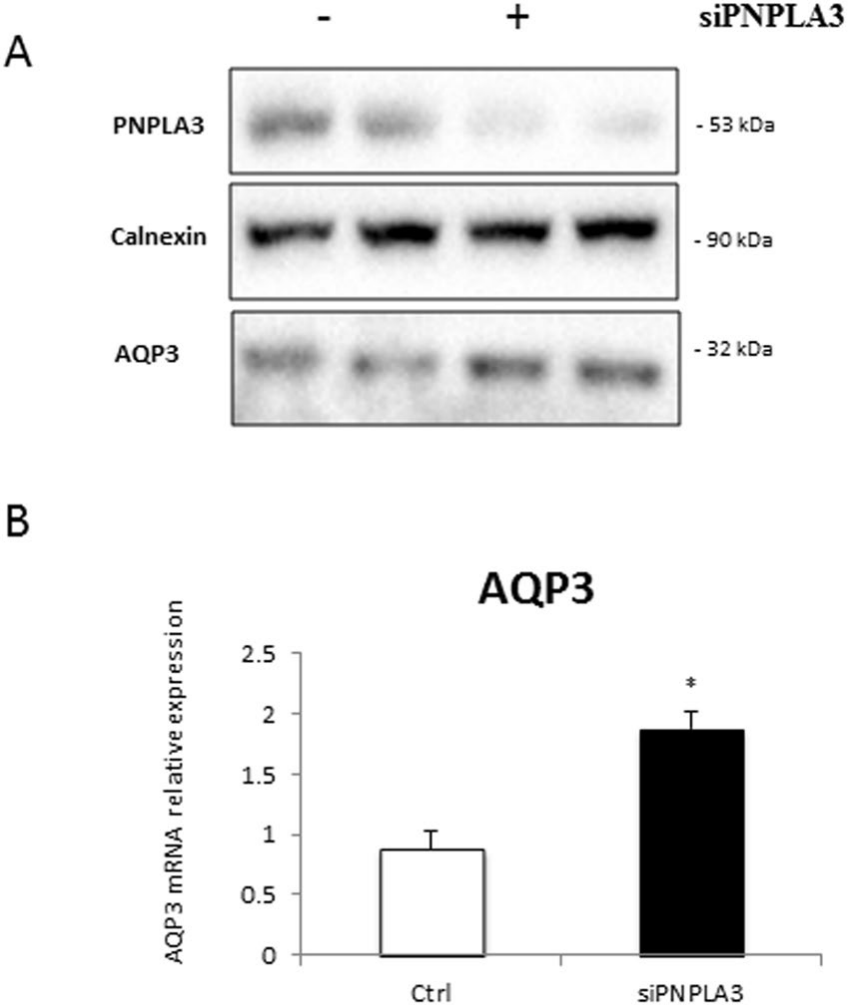
AQP3 expression upregulates after PNPLA3 silencing in LX2 cells. (**A**) Representative Western blot of PNPLA3 and AQP3 in LX2 cells treated with or without small interfering RNA (siPNPLA3), calnexin was used as a loading control. (**B**) AQP3 mRNA relative expression increased in LX2 cell lines silenced for PNPLA3 (siPNPLA3) versus control (Ctrl). (*p < 0.05).

**Figure 5:**
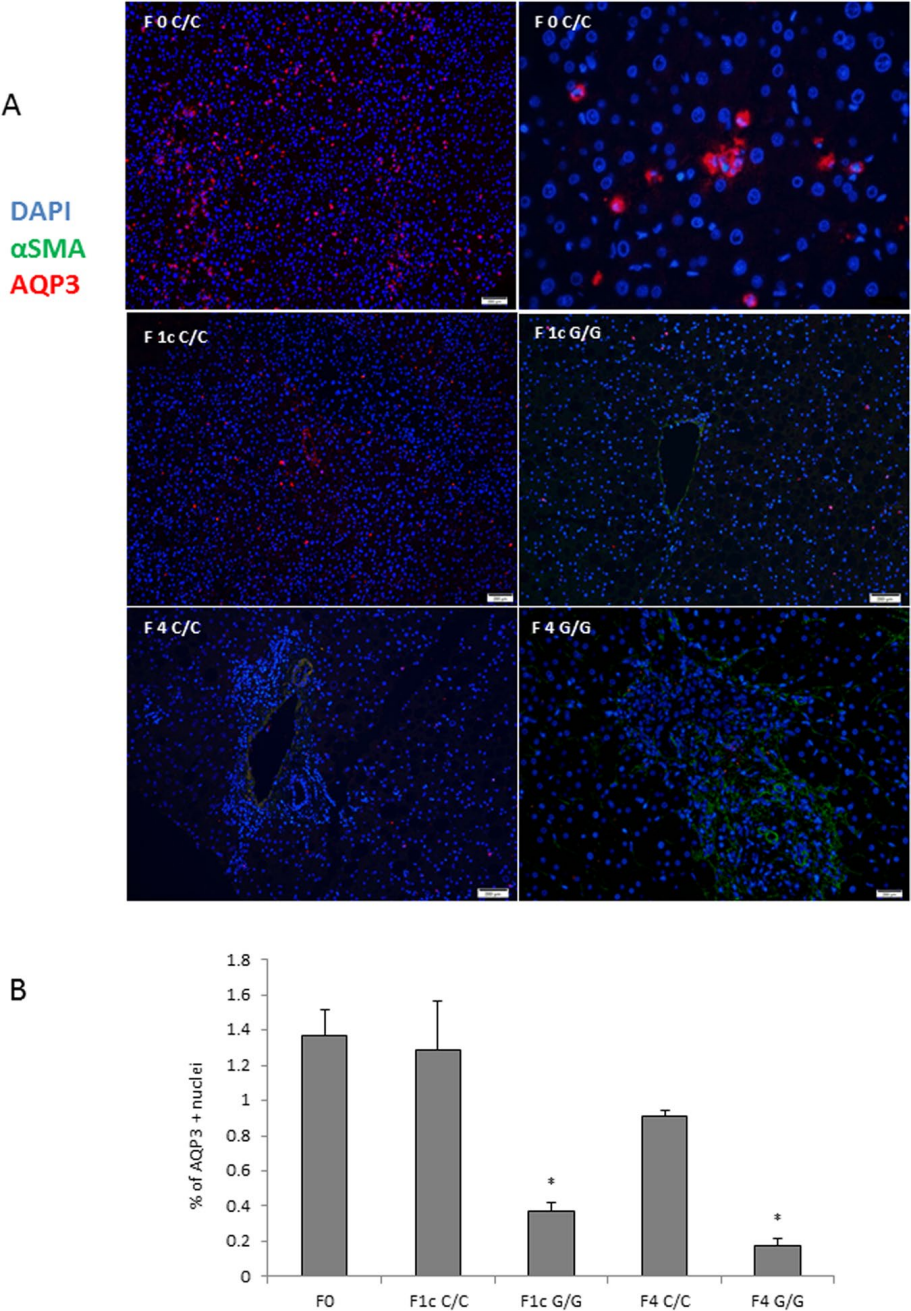
Immunohistochemistry of healthy (C/C) human liver showed more AQP3 abundance than PNPLA3 I148M (G/G) patients. (**A**) Representative images with merged immunofluorescence of AQP3 depicted in red, α-SMA depicted in green and Dapi in blue of control liver (C/C) and PNPLA3 I148M (G/G) at different stages of fibrosis (0, 1c, 4). Magnification all 20X, up panel right 40 X. (**B**) Relative quantification of AQP3 positive cells show G/G patients having overall less AQP3 than any stage of C/C (*p < 0.05; p values for F1c G/G vs F1c C/C: p = 0.04; F4 G/G vs F1c C/C p = 0.04). AQP3 + nuclei divided for the total amount of cells per slide, results of 5 pictures per slide shown as mean percentage ± SD of patients per fibrosis stage and genotype.

In order to further explore the dependence of AQP3 on PPARγ in HSC, we used LX2 overexpressing PNPLA3 wild type (WT) and I148M, since the latter have previously shown^8^ to lack PPARγ. As seen in Fig. 3A, AQP3 was present only in LX2 cells overexpressing PNPLA3 WT, with a dramatic down regulation in LX2 cells overexpressing the PNPLA3 I148M variant. Notably, RSG treatment of I148M PNPLA3 strongly restored AQP3 expression while WT cells displayed a relatively milder up-regulation of AQP3 (Fig. 3B). Conversely, when we stably silenced PNPLA3 in LX2 cells (Fig. 4A) AQP3 expression was strongly up-regulated (Fig. 4B). In order to explore the relevance for AQP3 in humans, we performed an immunofluorescence double staining of AQP3 and α-SMA of either healthy liver patient tissues (all fibrosis stage 0 and WT for PNPLA3, n = 4) or NASH patients with fibrosis stage 1c and 4 (n = 5 for PNPLA3 I148M, n = 5 for PNPLA3 WT). Our data showed that AQP3 is relatively more abundant in normal WT PNPLA3 liver (C/C) than in PNPLA3 I148M livers (G/G) (Fig. 5A,B) and inversely correlates with the stage of fibrosis. In conclusion, we demonstrated that PPARγ robustly up-regulates AQP3 expression in HSCs in a clear PNPLA3 isoform-dependent manner and that in line PNPLA3 I148M patients show lower AQP3 expression.

### AQP3 is regulated by PPARγ through JNK pathway and shows a tight dependence on PNPLA3

**Figure 6:**
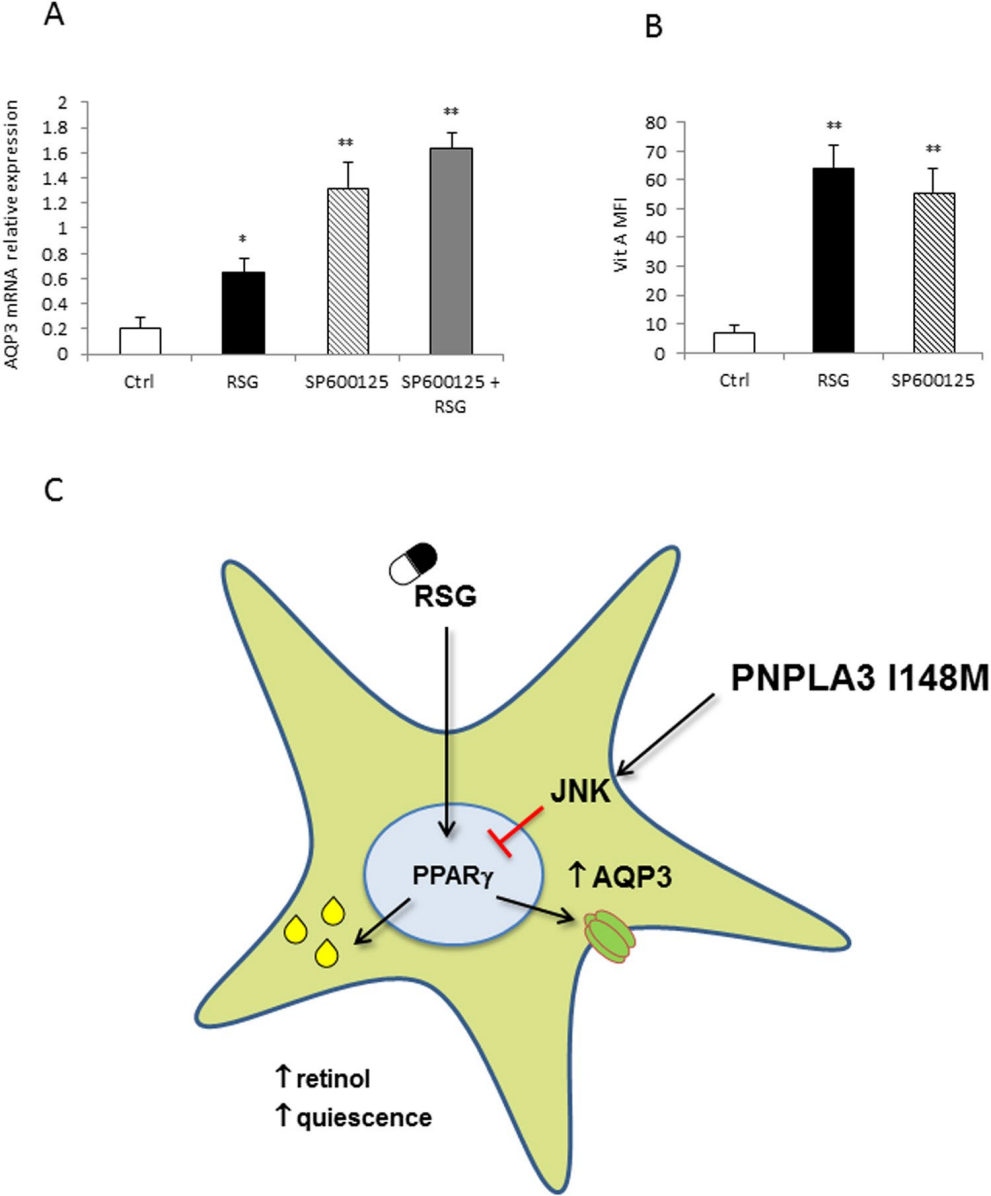
The JNK antagonist SP600125 increased AQP3 expression and Vitamin A content in LX2 cells overexpressing PNPLA3 I148M, showing an additive effect when combined with rosiglitazone. (**A**) AQP3 mRNA relative expression increased after treatment with a JNK inhibitor (SP600125) and rosiglitazone (RSG) compared to untreated cells (Ctrl) (*p < 0.05, **p < 0.01). (**B**) Vitamin A cells´ content increased after same treatments in a flow cytometric analysis (**p < 0.01 vs untreated control - ctrl), with representative dot plots shown in Suppl. Figure 2. C. Schematic summary of the proposed molecular mechanism. RSG increases PPARγ level, which in turn regulates AQP3 expression and glycerol shuttling. PNPLA3 I148M activates JNK pathway, which suppresses PPARγ and consequently AQP3 expression.

Since we previously demonstrated that PNPLA3 I148M activates JNK pathway via blocking PPARγ action, which could be partially reversed by a PPARγ agonist^8^, we next explored whether blockade of JNK pathway with a specific antagonist (SP600125) affected AQP3 expression. LX2 cell line overexpressing PNPLA3 I148M treated with JNK antagonist showed increased AQP3 expression with an additive effect in combination with RSG (Fig. 6A). Moreover, cells with inhibited JNK pathway had higher vitamin A content (Fig. 6B, Suppl. Figure 2), cell size and subsequently became quiescent as reflected by loss of p21 (not shown). Collectively as summarized schematically in Fig. 6C, the impact of PNPLA3 I148M on AQP3 expression could be blocked by JNK inhibition and PPARγ stimulation.

## Discussion

Aquaporins are interesting but extremely elusive targets in human physiology^22^. They are expressed in many organs but their metabolic function, especially in adipose tissue and liver, is still unclear^14,22–24^. AQP9 in hepatocytes has been by far the most studied AQP in liver^25–27^, being a key player in the development of liver steatosis^28^. AQP3 expression was originally found in colon, kidney and liver in humans and had *in vitro* the capacity to transport water, urea and glycerol^29,30^. While AQP3 deletion in kidney resulted in polyuria^31^, deletion in skin reduced glycerol and water content^32^ showing that *in vivo* AQP3 transports water as well as glycerol. Despite its hepatic expression, AQP3 cell type localization in the liver was not investigated so far. In HSC, a few studies from our group^33,34^ explored the metabolic relevance of AQP3 in liver fibrosis and its tight hormonal regulation by adiponectin. However, our experiments took place only in LX2 cell line, therefore some questions remained open on the pathway involved and its meaning *in vivo*. We tested AQP3 expression in human primary HSC, discovering the molecular mechanism involved also in connection to a disease model.

The PNPLA3 I148M variant is key risk factor for development of hepatic steatosis and its progression to more severe liver disease, with development of more advanced fibrosis, cirrhosis and cancer^11,35,36^. A recent study from our group^8^, uncovered how PNPLA3 I148M in HSC results in an intrinsic reduction of PPARγ, the main gene involved in lipid metabolism and AQPs regulation^21,25,27^. Importantly, several AQPs have been shown to be regulated by PPARγ agonists. More specifically, RSG induced AQP7 expression in adipose tissue from OLETF rats and AQP3 in a hepatoma cell line^37^. In line, in leptin deficient mice treated with RSG, the levels of AQP3 in subcutaneous adipocytes and AQP9 in hepatocytes increased^38^. Therefore, we aimed to explore a putative connection between AQP3 expression and PNPLA3 I148M within HSC activation.

We here demonstrate that AQP3 is the only aquaporin expressed in human primary HSC consistent with previous findings in LX2 cells^33^, and repressed during their activation. Treatment with rosiglitazone, a PPARγ agonist and upstream transcription factor known to regulate AQPs expression in other organs^20^, induced AQP3 expression and therefore may modulate glycerol influx. In line with reduced PPARγ activity in PNPLA3 I148M^8^, AQP3 expression decreased dramatically. This highlights the interplay between AQP3 and PNPLA3, consistent with their roles in glycerol uptake and triglyceride synthesis or degradation, respectively, further emphasized by the increased expression of genes involved in lipogenesis. Importantly, downregulation of AQP3 in PNPLA3 I148M could be strongly counteracted with the PPARγ agonist rosiglitazone in comparison to WT cells, underlying a strong PPARγ dependence. Moreover when PNPLA3 was silenced in LX2 cells, AQP3 strikingly upregulated. These data indicate that PPARγ is not only required for stimulation of AQP3, but may also drive its baseline expression. Interestingly, PPARγ agonists are known to promote white adipose tissue differentiation and expansion in pre-adipocytes and stimulate lipogenesis^39^, similar to what we now observed in HSC. In human healthy liver AQP3 is also widely present, however its abundance decreased with fibrosis progression, and was also dependent on PNPLA3 mutation. This was in line with another previous report^16^ in which AQPs expression decreased in liver of ethanol/lipopolysaccharide fibrotic animal model.

A study from our group recently demonstrated an up-regulation of the JNK/AP-1 pathway in PNPLA3 I148M cells, which could be partially counteracted by rosiglitazone treatment^8^. Notably, PPARγ itself suppresses JNK activation via directly binding Jun D and downregulating AP-1^12^. Since others showed a down-regulation of AQP3 in keratinocytes after UV radiation, another JNK activator^40,41^ and that PPARγ blocks JNK pathway through direct binding to Jun D^12^, we used a JNK inhibitor to explore the impact of this pathway on AQP3 regulation in HSC. Indeed, although others showed the involvement of PI3K/Akt/mTor pathway in AQPs regulation in adipocytes and hepatocytes^42^, in HSC this appears to occur through JNK. Moreover, JNK inhibition also increased the amounts of vitamin A and lipids in the cells, indicating that reduction of AQP3 may disrupt glycerol metabolism in HSC.

PPARs were shown to be promising targets for treatment of fibrosis; in details, a PPARγ agonist like pioglitazone has been shown to increase AQPs expression^21^, ameliorate insulin resistance inducing adipogenesis, and a local effect in HSC should not be underestimated as it renders them quiescent. Moreover, it was recently demonstrated pioglitazone antifibrotic effects in a cohort of human diabetics with NASH^43^. A long term treatment, up to 3 years, was valued safe, well tolerated with no significant drug-related side effects; pioglitazone treatment showed to improve liver steatosis, inflammation and ballooning scores, also ameliorating metabolic and histological parameters in NASH, pre-diabetic and type 2 diabetic patients^43^.

In conclusion, AQP3 is regulated in PNPLA3 I148M via the JNK pathway through a PPARγ mediated activation and associates to different degrees of fibrosis. Future studies are required to decipher whether AQPs are targetable within HSCs activation, in order to develop new treatments for liver fibrosis in PNPLA3 I148M patients.

## Materials and Methods

### Isolation and culture of primary human HSCs and LX-2

HSCs were isolated from surgical liver resections unsuitable for transplantation, all experimental protocols were approved by the Ethics Committee of Medical University of Florence^44^ after informed consent from all subjects, and carried out in accordance with relevant guidelines and regulations. After mechanical digestion, a multi-step enzyme digestion with a collagenase/pronase/DNAase^44^ solution was performed. Hepatic cells were centrifuged and separated using a density gradient (Percoll, GE Amersham, Arlington Heights, IL). HSCs were seeded on uncoated plastic dishes and cultivated with Iscove’s Modified Medium (Dulbecco’s medium, EuroClone, Italy) supplemented with 20% non-heat inactivated fetal bovine serum, 0.2 mol/L glutamine, sodium pyruvate 0.1 mol/L, non-essential amino acid solution 100x, antibiotic, antimycotic solution 100x (Life Technologies, Carlsbad). After 5 days of culture the purity was ~95% as estimated by retinoid auto fluorescence. All experiments with primary HSCs were performed on cells from passage 1 to 8. Primary HSCs at the same passage were used for comparison experiments of WT and I148M PNPLA3. The genotype of each isolated HSC line has been analyzed by real-time PCR for the I148M SNP, as done routinely in our lab^8^. LX-2 cell line, kindly provided by Prof. S.L. Friedman (Mount Sinai School of Medicine, NY), an *in vitro* model of partially activated HSCs^45^, were cultured in Dulbecco’s modified Eagle’s medium (DMEM) with 4.5 g/L glucose supplemented with 5% FBS, L-glutamine (0.2 mol/L) and antibiotics (all Thermo Fisher Scientific). The genotype of LX-2 cells was analyzed by real-time PCR for the I148M SNP^8^.

### Stable PNPLA3 wild type, I148M transfection and PNPLA3 silencing

LX-2 cells were transfected with 15 nanograms of pcDNA™3.1/V5-His-TOPO® (Thermo Fisher Scientific) carrying either the PNPLA3 wild type (WT) and I148M sequence as previously reported^8^. Silencing was performed with Lipofectamine® 2000 Transfection Reagent (Thermo Fisher Scientific) according to manufacturer’s instructions. The efficiency of transfection was evaluated via RT-quantitative PCR and western blotting^8^.

### RNA extraction and quantitative reverse transcriptase polymerase chain reaction (qRT-PCR) analysis

Primary HSC and LX2 cells were homogenized in TRIzol reagent (Thermo Fisher Scientific) and RNA was isolated according to the manufacturer´s protocol. Total RNA (1 µg) was transcribed into cDNA using Superscript II and random hexamer primers (Thermo Fisher Scientific). Gene expression of human *AQP3* (NM_004925.4), *AQP7* (NM_001170.2), *AQP9* (NM_001320635.1), *AQP10* (XM_011510104.2), *FASN* (NM_004104.4), *SCD1* (NM_005063.4), *PNPLA3* (NM_025225.2), *p21* (NM_000389.4), *PPARg* (NM 005037.5) and *SREBP1c* (NM_001321096.2) (all Thermo Fisher Scientific) was analyzed by quantitative real-time PCR on an ABI Step One Plus cycler using assays-on-demand kits (TaqMan® Gene Expression Assay, Thermo Fisher Scientific). Each reaction was performed in duplicates and the value of the gene of interest was normalized to human ubiquitin C expression. The comparative threshold cycle (CT) method was used to calculate the relative expression^46^.

### Western Blotting

Approximately, 500.000 cells per condition were collected in RIPA buffer (Radio immune precipitation assay buffer, 0.01 mol/L Tris-Cl (pH 8.0), 0.001 mol/L EDTA, 5 × 10^−4^ mol/L EGTA, 0.1% sodium deoxycholate, 0.1% SDS, 1% NP-40) and protein concentration was measured using 660 nm protein assay kit. Twenty micrograms per sample were loaded on a SDS-PAGE using 10% polyacrylamide gels. α-SMA was identified using a 1:2000 dilution of the monoclonal mouse anti-humanα-SMA (Sigma-Aldrich), AQP3 was detected using a 1:500 dilution of monoclonal rabbit anti-AQP3 as performed before^47^. PNPLA3 was detected with a 1:1000 dilution of the rabbit polyclonal anti-PNPLA3 (Abcam, Cambridge, UK). Band intensity achieved from these antibodies was normalized to band intensity of Calnexin using mouse anti-calnexin 1:3000 (Santa Cruz Biotechnology Inc, Dallas, TX, USA).

### Flow cytometry

HSC (approximately 70.000 cells/well) were incubated with the primary antibody (Rabbit anti-AQP3, Sigma Aldrich), diluted 1:500 in blocking solution, for 45 mins at RT, followed by washing and incubation with a fluorochrome-labelled secondary antibody (Alexa Fluor 594 – goat anti rabbit, Thermo Scientific) diluted 1:500 in the blocking solution for 1 h in the dark. Cells were washed twice in PBS and prepared for flow cytometric analysis. Retinol amount contained in LX2 cells was analysed exploiting its intrinsic autofluorescence with an 351 nanometers excitation as seen^48^. Flow cytometry was performed with BD FACSCanto™ II and BD FACSDiva™ software (Becton Dickinson New Jersey, USA).

### Immunohistochemistry

Healthy specimens were collected after liver resection of colorectal metastasis (n = 4), whereas fibrosis samples (n = 10) were obtained by percutaneous liver biopsy (informed consent was obtained from all subjects); the study and all experimental protocols were approved by the local ethics committee (EK747/2011) and carried out in accordance with relevant guidelines and regulations, samples were evaluated by a board certified pathologist. Formalin-fixed human liver slides were de-paraffinized and prepared for hematoxylin & eosin staining. For the immunofluorescence, slides were blocked for 1 h in blocking buffer (1XPBS, 5% goat serum and 0.3% Triton™ X-100) as seen previously^49^. Blocking buffer was discarded and sections incubated overnight with polyclonal rabbit anti-human AQP3 antibody (Sigma-Aldrich) diluted 1:250 in PBS with 5% goat serum (Dako, Glostrup Municipality, Denmark) at 4 °C. Slides were washed three times in PBS and incubated for 1 h at room temperature with a 1:500 dilution of monoclonal mouse anti-human α-SMA antibody (Sigma-Aldrich). Slides were then washed and incubated for 1 h in the darkness with the secondary antibody Alexa Fluor 594 goat anti rabbit IgG (1:500, Thermo Scientific) for AQP3. Slides were washed three times in PBS and the process was repeated with Alexa Fluor 488 goat anti mouse IgG (1:500, Thermo Scientific) for α-SMA. Thereafter, nuclei were counterstained with DAPI (Sigma) for 10 min, washed and mounted (VECTASHIELD® Mounting medium) for microscope analysis (Olympus BX51). The relative amount of AQP3 + cells was quantified and divided by the amount of nuclei per field. A number of 5 pictures were taken for each liver slide, and the average was representative for one patient as already performed in^49^.

### Statistics

Data are presented as mean ± standard deviation of 3 independent experiments performed in duplicates. Kruskal-Wallis test was employed for non-parametric multi-group comparisons, Mann-Whitney U test for comparisons between two groups only. A P-value < 0.05 was considered statistically significant. All statistics were calculated using SPSS 22.0 software (Chicago, IL, USA).

### Data availability

All data generated or analysed during this study are included in this published article (see supplemmatry material for original Western blots).

## Electronic supplementary material

Supplementary Material
