# Supplementary material for: AQP3 is regulated by PPARγ and JNK in hepatic stellate cells carrying PNPLA3 I148M
Source: Sci Rep. 2017 Nov 7;7:14661. doi: 10.1038/s41598-017-14557-9 (PMC5676689; doi:10.1038/s41598-017-14557-9)
Supplement: Supplementary file 1 — Supplementary Material [file 41598_2017_14557_MOESM1_ESM.doc]

***Supplementary Material:***

**AQP3 is regulated by PPARγ and JNK in hepatic stellate cells carrying PNPLA3 I148M**

Matteo Tardelli (1), Francesca V. Bruschi (1), Thierry Claudel (1), Veronica Moreno-Viedma (2)(3), Emina Halilbasic (1), Fabio Marra (4), Merima Herac (5), Thomas M. Stulnig (2) and Michael Trauner (1)*

(1) Hans Popper Laboratory of Molecular Hepatology, Division of Gastroenterology & Hepatology, Internal Medicine III, Medical University of Vienna, Austria

(2) Christian Doppler-Laboratory for Cardio-Metabolic Immunotherapy and Clinical Division of Endocrinology and Metabolism, Department of Medicine III, Medical University of Vienna, Austria.

(3) Institute of Cancer Research, Department of Medicine I, Comprehensive Cancer Center, Medical University of Vienna, Austria.

(4) Clinical Pathophysiology Department, Medical University of Florence, Italy.

(5) Clinical Institute of Pathology, Medical University of Vienna, Austria.

Correspondence:

Michael Trauner, MD

Division of Gastroenterology and Hepatology, Department of Internal Medicine III, Medical University of Vienna, Währinger Gürtel 18-20, A-1090, Vienna, Austria.

Tel.: +43 1 40 40047410, Fax: +43 1 404004735

E-mail: [michael.trauner@meduniwien.ac.at](mailto:michael.trauner@meduniwien.ac.at)

**Supplementary Fig. 1**. Representative dot plots and gating of flow cytometric indirect staining for AQP3, referred to Fig. 1 E.

**HSC D1**


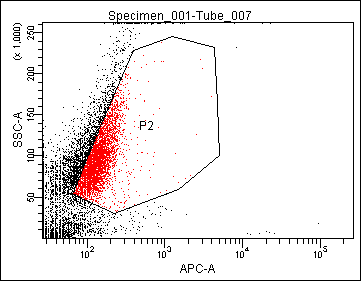

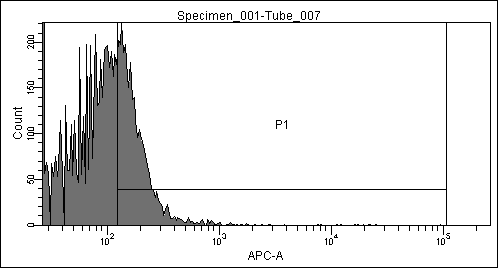

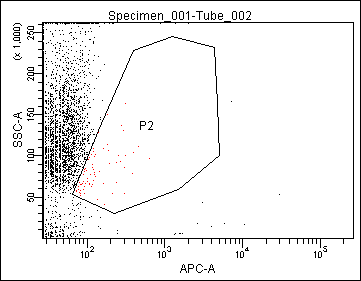

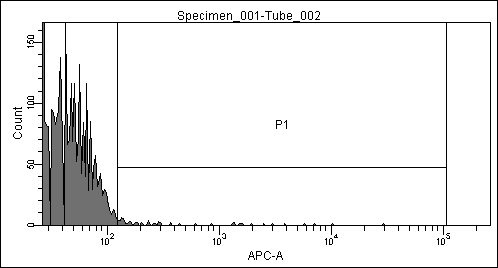

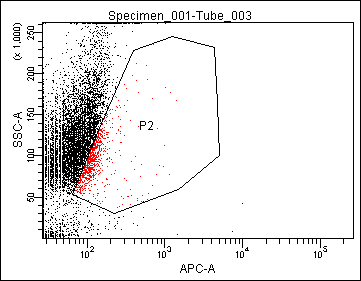

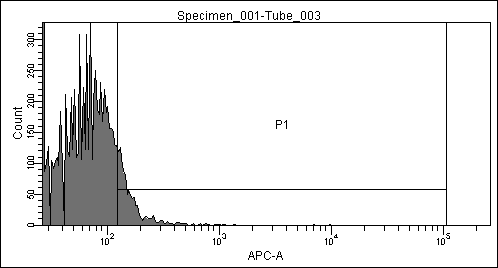


**HSC D3**

**HSC D7**

**Supplementary Fig. 2.** Representative dot plots and gating of flow cytometric analysis of vitamin A content, referred to Fig. 6 B.

**
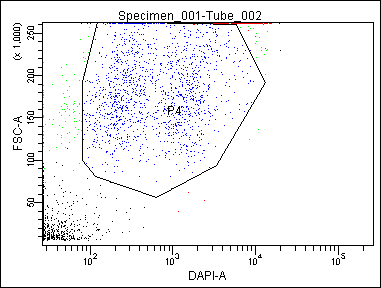

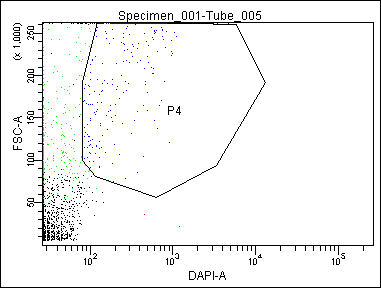

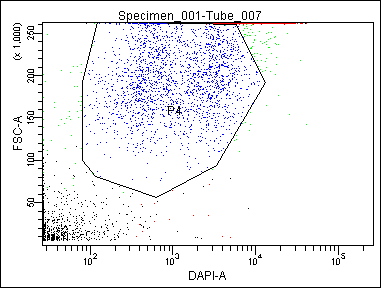
**

**Ctrl**

**RSG**

**SP600125**

**Supplementary Fig. 3 –** original blots

Original blots for Fig. 1C showing Calnexin, α-SMA, AQP3.


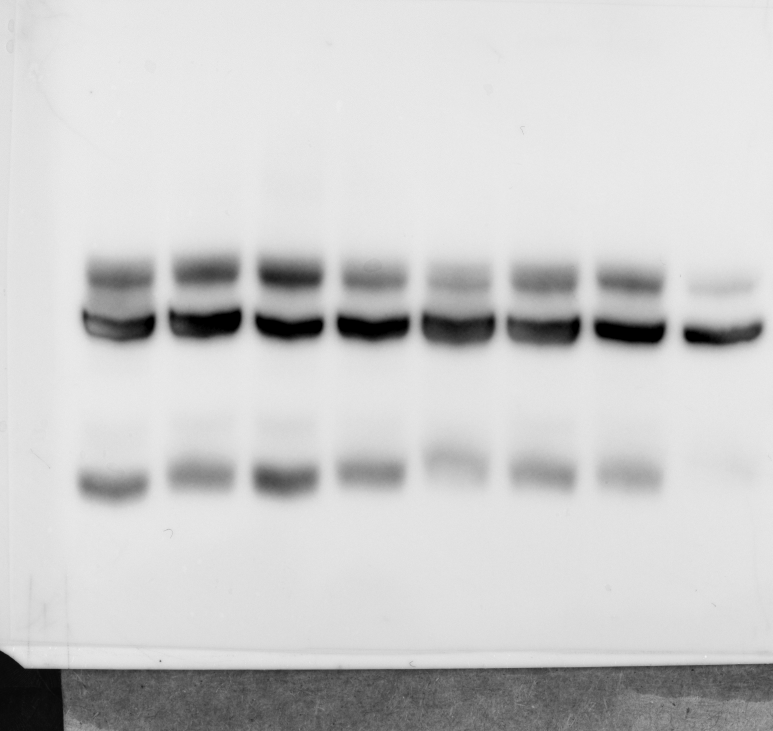


**Calnexin**

**AQP3**

**D1**

**D3**

**D7**


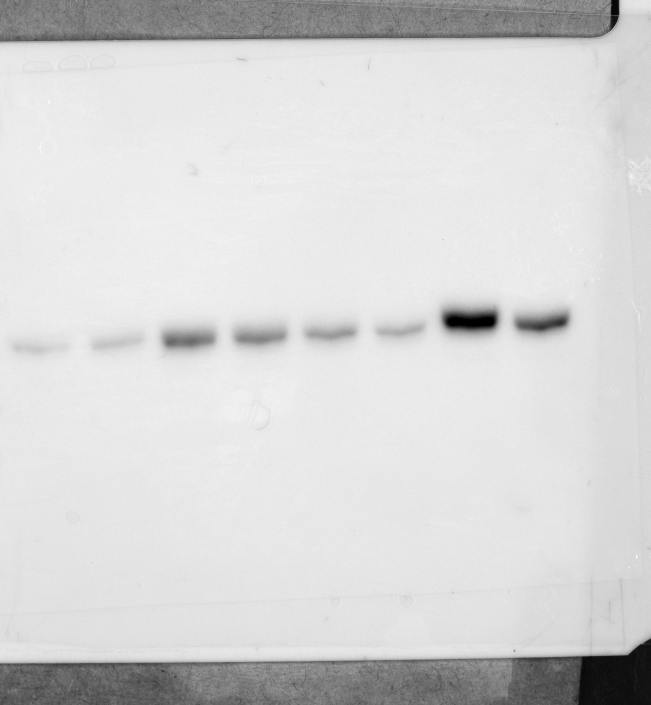


**α-SMA**

**D1**

**D3**

**D7**

Original blots for Fig. 3A showing AQP3, α-SMA and Calnexin.


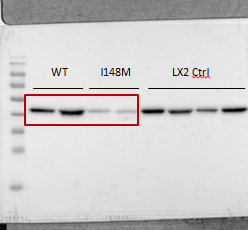


**AQP3**


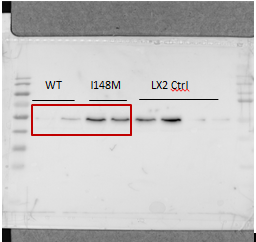


**α-SMA**


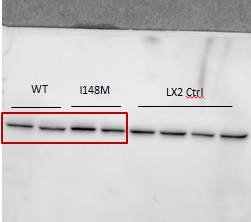


**Calnexin**

Original blots for Fig. 4A showing Calnexin, PNPLA3 (in two different exposures, 20, 10 sec) and AQP3, red boxes represent the lanes shown in the main figure.

**siPNPLA3**

- +

+

Ctrl

-

-


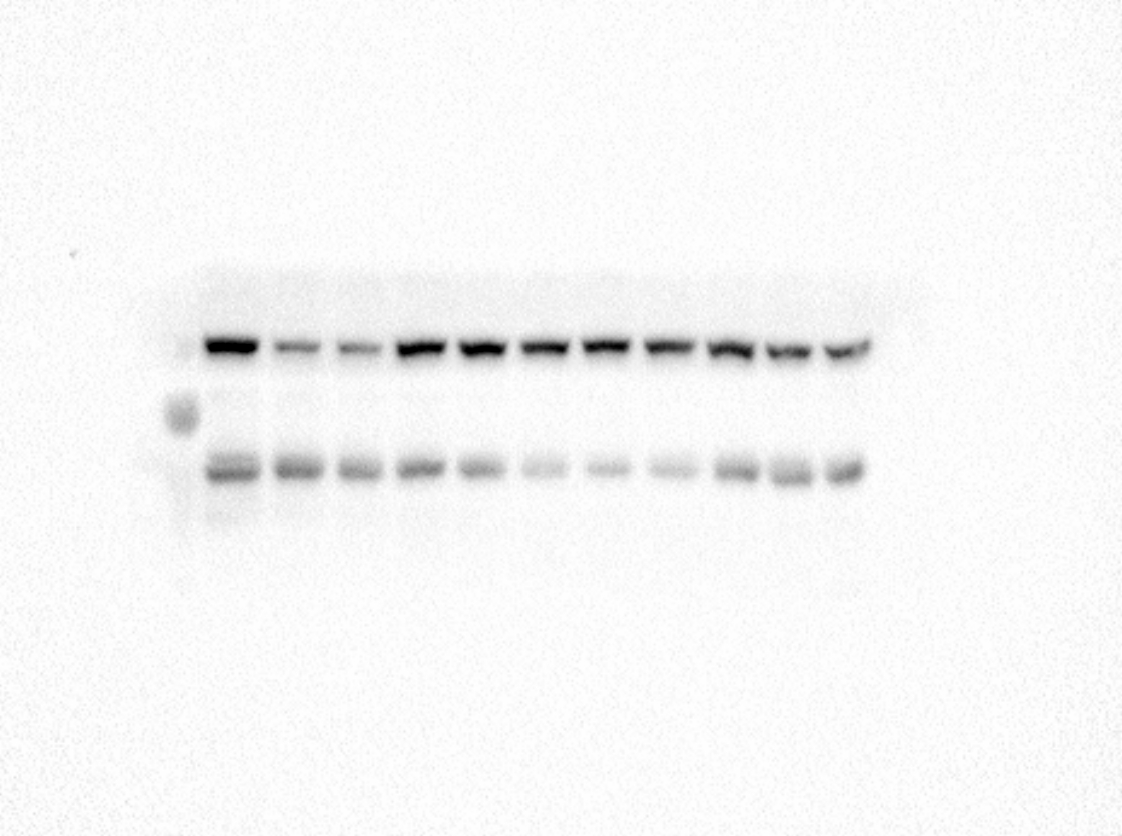

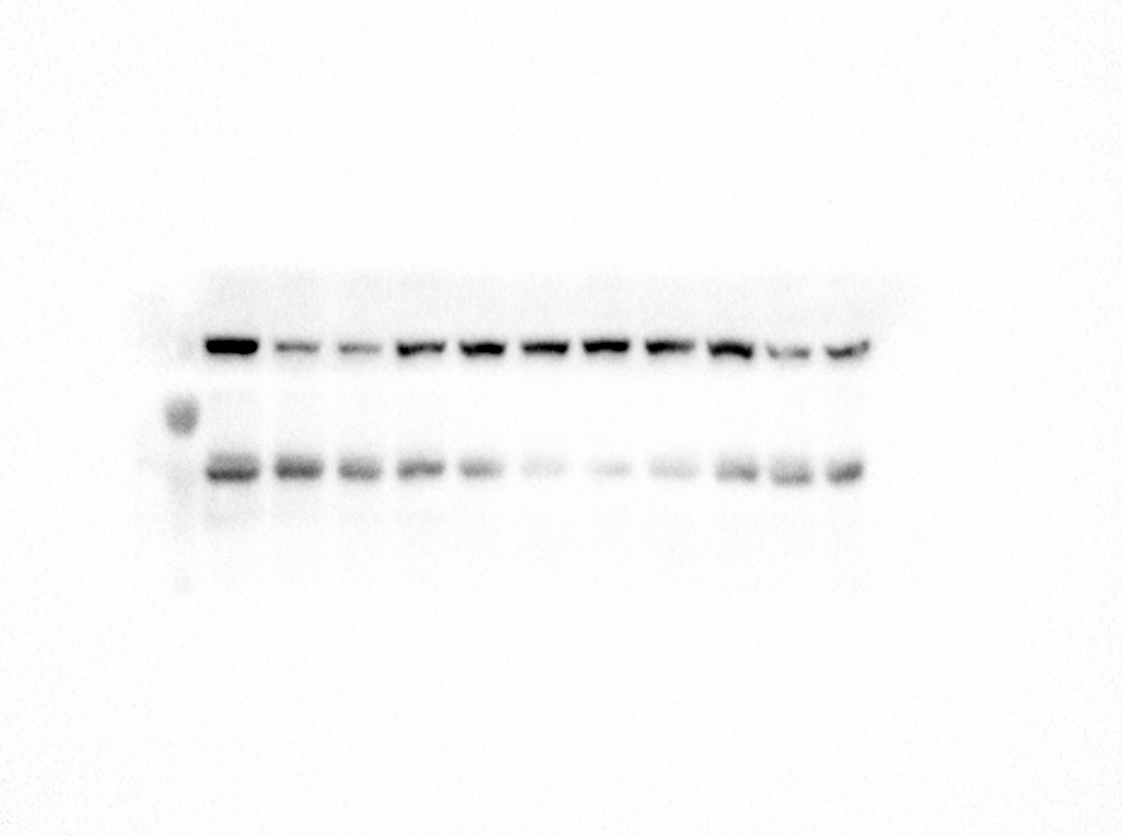


**Calnexin**

**PNPLA3**

**Calnexin**

**PNPLA3**

**siPNPLA3**

- +

- +

**siPNPLA3**

Ctrl

-

Ctrl

-

+

-

+

-


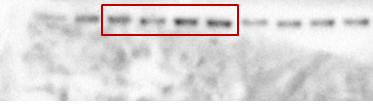


**AQP3**
